# Incorporation of DPP6a and DPP6K Variants in Ternary Kv4 Channel Complex Reconstitutes Properties of A-type K Current in Rat Cerebellar Granule Cells

**DOI:** 10.1371/journal.pone.0038205

**Published:** 2012-06-04

**Authors:** Henry H. Jerng, Paul J. Pfaffinger

**Affiliations:** Department of Neuroscience, Baylor College of Medicine, Houston, Texas, United States of America; Georgia State University, United States of America

## Abstract

Dipeptidyl peptidase-like protein 6 (DPP6) proteins co-assemble with Kv4 channel α-subunits and Kv channel-interacting proteins (KChIPs) to form channel protein complexes underlying neuronal somatodendritic A-type potassium current (I_SA_). DPP6 proteins are expressed as N-terminal variants (DPP6a, DPP6K, DPP6S, DPP6L) that result from alternative mRNA initiation and exhibit overlapping expression patterns. Here, we study the role DPP6 variants play in shaping the functional properties of I_SA_ found in cerebellar granule (CG) cells using quantitative RT-PCR and voltage-clamp recordings of whole-cell currents from reconstituted channel complexes and native I_SA_ channels. Differential expression of DPP6 variants was detected in rat CG cells, with DPP6K (41±3%)>DPP6a (33±3%)>>DPP6S (18±2%)>DPP6L (8±3%). To better understand how DPP6 variants shape native neuronal I_SA_, we focused on studying interactions between the two dominant variants, DPP6K and DPP6a. Although previous studies did not identify unique functional effects of DPP6K, we find that the unique N-terminus of DPP6K modulates the effects of KChIP proteins, slowing recovery and producing a negative shift in the steady-state inactivation curve. By contrast, DPP6a uses its distinct N-terminus to directly confer rapid N-type inactivation independently of KChIP3a. When DPP6a and DPP6K are co-expressed in ratios similar to those found in CG cells, their distinct effects compete in modulating channel function. The more rapid inactivation from DPP6a dominates during strong depolarization; however, DPP6K produces a negative shift in the steady-state inactivation curve and introduces a slow phase of recovery from inactivation. A direct comparison to the native CG cell I_SA_ shows that these mixed effects are present in the native channels. Our results support the hypothesis that the precise expression and co-assembly of different auxiliary subunit variants are important factors in shaping the I_SA_ functional properties in specific neuronal populations.

## Introduction

The somatodendritic A-type potassium current (I_SA_) regulates neuronal excitability, firing frequency, action potential back-propagation, and synaptic plasticity [1], [2], [3], [4]. Features of I_SA_ essential for its function include rapid activation, inactivation, and recovery from inactivation all of which are dynamically modulated at sub-threshold membrane potentials [5]. Inactivation is a particularly important gating property to understand in I_SA_ channels since the midpoint of steady-state inactivation is typically near a neuron's resting membrane potential. Because of this, small membrane potential variations from rest can produce significant changes in functional levels of I_SA_ in a fraction of a second, and consequently the neuron can dynamically adjust the level of I_SA_ in response to prevailing conditions. In addition, the kinetic properties of inactivation gating also contribute to how this regulatory process plays out as it determines how the pattern of voltage fluctuations occurring around the resting potential are translated into different levels of channel inactivation.

I_SA_ channels are multi-protein conglomerates composed of Kv4 type voltage-gated potassium channel subunits (Kv4.1, Kv4.2, Kv4.3) surrounded by modulatory subunits from two distinct gene families: Kv channel-interacting proteins (KChIPs) and dipeptidyl peptidase-like proteins (DPLPs) [5], [6], [7]. KChIPs are cytoplasmic calcium-binding proteins that bind to both the N-terminus and the tetramerization (T1) domain of Kv4 channel subunits, thereby occluding the endogenous fast inactivation mediated by the Kv4 N-terminus, promoting surface expression by masking an ER-retention signal, and accelerating recovery from inactivation [8], [9], [10], [11]. The four KChIP genes (KChIP1, KChIP2, KChIP3, KChIP4) express alternative transcripts that encode distinctive KChIP N-terminal variants, some of which are known to mediate unique effects [5], [12]. DPLPs are single-pass, type II transmembrane proteins with a short N-terminal intracellular segment and a large extracellular domain [13], [14]. The transmembrane domain of DPLPs interacts with the voltage sensing portion of the Kv4 subunits [15], mediating accelerated activation, inactivation, and recovery from inactivation along with hyperpolarizing shifts in the voltage dependence of steady-state activation and inactivation [16], [17], [18]. The two genes in the DPLP family, dipeptidyl peptidases 6 (DPP6) and 10 (DPP10), also produce distinctive transcripts from multiple promoter sites which encode different N-terminal protein variants (DPP6: DPP6a/DPP6E, DPP6K, DPP6L, DPP6S; DPP10: DPP10a, DPP10b, DPP10c, DPP10d) [19], [20], [21], [22]. Unique functional effects have been described for DPP6a and DPP10a due to a conserved N-terminal sequence (MNQTA) that induces exceptionally fast voltage-independent inactivation through an N-type pore-blocking mechanism [22]. Strong evidence for I_SA_ channels being composed of a ternary complex of Kv4s, KChIPs, and DPLPs comes from co-immunoprecipitation and functional reconstitution experiments demonstrating that native-like functional properties requires co-expression of all three proteins [16], [23]. In agreement with this model, the transcript and protein expression patterns of Kv4, KChIP, and DPLPs overlap in all regions of the brain, suggesting an obligatory ternary complex formation underlies I_SA_ throughout the brain [24], [25].

Cerebellar granule (CG) cells are an ideal neuronal population to investigate the role of DPP6 variants in shaping native I_SA_ properties. CG cells are electrophysiologically compact and express large I_SA_. DPP6 is the only DPLP that is expressed in CG cells [19], [26], [27], and DPP6 is required for normal I_SA_ channel formation and function [28]. Previous studies have suggested possible roles for DPP6S and DPP6a in shaping CG cell I_SA_; however, a proper measurement of the expression levels of different DPP6 variants in CG cells is lacking, as are the consequences of DPP6 variant co-expression for shaping I_SA_ properties [26], [29].

In this study, we sought to determine how expression of multiple DPP6 variants shapes the various functional properties of the native I_SA_ in CG cells. Our results show that the DPP6K variant constitutes the largest percentage of total DPP6 expressed in CG cells, followed by DPP6a, DPP6S, and DPP6L. Because of its large contribution to DPP6 transcripts, DPP6K was re-examined in co-expression studies with Kv4.2 and KChIP3a, and we found that in the heterotrimeric complex DPP6K does generate unique functional effects that were not seen in previous co-expression studies with Kv4.2 alone [19]. Co-expression of DPP6K in the ternary complex results in inactivation kinetics that is faster than seen with DPP6S, and a hyperpolarizing shift in steady-state inactivation with significantly slower recovery from inactivation. Since multiple DPLP proteins can co-assemble into a single channel [30], we wanted to test if the co-expression of multiple DPP6 variants can lead to I_SA_ channels with unique functional properties. Detailed biophysical analysis shows that co-expression of DPP6a and DPP6K at 1∶2 ratio with Kv4.2 and KChIP3a generates transient currents with unique functional parameters that are a close match for I_SA_ recorded from CG cells. In particular, the addition of DPP6K accelerated the slow phase of inactivation and introduced a slow phase of recovery from inactivation, both detected with the native I_SA_. Substituting DPP6K with DPP6S decreased the similarity between reconstituted and native currents, showing that the functional properties of DPP6K are critically important for correctly forming native I_SA_ in CG cells. In summary, our results show that (1) DPP6K is the most common DPP6 variant expressed in CG cells, (2) DPP6K N-terminal domain regulates the slower phases of inactivation, recovery from inactivation, and steady-state inactivation of the ternary complex, and (3) The properties of the native I_SA_ in CG cells are largely determined by the co-assembly of Kv4.2, KChIP3a, DPP6a, and DPP6K subunits into a heteromultimeric channel complex.

## Materials and Methods

### Ethics Statement

The procedures on animals conducted in this work were performed in strict accordance with Animal Welfare Act, the Public Health Services Animal Welfare Policy, and The National Institute of Health Guide for Care and Use of Laboratory Animals. The experimental protocol was approved by the Institutional Animal Care and Use Committees (IACUC) of Baylor College of Medicine (Protocol Number: AN-752). Following approved protocol, every effort was made to minimize suffering.

### Tissue Procurement and Preparation of Brain Slices for *In Situ* Hybridization and Slice Recordings

Sprague-Dawley rats were anesthetized by isoflurane inhalation (IsoFlo, Abbott Laboratories), rapidly killed by cervical dislocation, and decapitated using a guillotine. Brain tissues from P21–28 rats were used for synthesizing cDNA used in amplification PCR or qRT-PCR. For *in situ* hybridization, the whole brains of P12 rats were removed, frozen in isopentane, and sliced into 20 µm sections using a Jung CM3000 cryostat as previously described [20]. The sections were mounted on poly-L-lysine-coated slides and processed for probing using rabio-labelled riboprobes.

For slice recordings, the whole brain from young P8–10 rats was divided using a blade into the two hemispheres and cerebellum. The blocks of tissue were immersed immediately in normal artificial cerebrospinal fluid (ACSF) (in mM: 124 NaCl, 44 KCl, 2 CaCl_2_, 1.2 MgSO_4_, 1 NaH_2_PO_4_, 2.6 NaHCO_3_, 1 glucose) saturated with 95% O_2_/5% CO_2_ at room temperature (RT, 21–23°C). After 30 min, a small block of cerebellar tissue is properly oriented and adhered to the cutting tray of the vibratome (VT1000S, Leica Microsystems Inc.) using cyanoacrylate glue (Vetbond, 3M), and the tray is submerged in ice-cold cutting solution (in mM: 182.6 sucrose, 30 NaCl, 4.5 KCl, 1 MgCl_2_, 1.2 NaH_2_PO_4_, 1 NaHCO_3_, 1 glucose). Cerebellar slices (300 µm) were cut in the coronal plane by the vibratome's automated function and stored at room temperature on a mesh submerged under normal ACSF saturated with 95% O_2_/5% CO_2_. After a 1 hr recovery period, a slice was transferred to the recording chamber perfused with normal ACSF saturated with 95% O_2_/5% CO_2_ at RT.

### Molecular Biology

A 5′RACE-PCR was conducted on RNA isolated from the cerebellum of a p28 rat using the First Choice RLM RACE Kit (Ambion) as described previously [20]. The DPP6a-specific probe used in *in situ* hybridization was amplified from the 5′ RACE-PCR products, using the following PCR primers: (f) 5′-TGCTCTAGAGAGCTCACAGAAGCTTGGAGTACAG-3′ and (r) 5′-CCGGAATTCCTTATGGCCTTTCCCTGGAGGACAC-3′, where the underlined nucleotides represent nested XbaI and EcoRI sites in forward and reverse primers, respectively. The probe was digested with XbaI and EcoRI and subcloned into pBluescript II KS(+) (Stratagene) to generate pBS/DPP6a-ribo. Radio-labeled antisense and sense riboprobes were synthesized from pBS/DPP6a-ribo linearized by EcoRI and XbaI, respectively, using the Riboprobe In Vitro Transcription Systems (Promega Corporation) in the presence of [^35^S]-UTP (Perkin Elmer Life and Analytical Sciences).

To generate the normalization controls for qRT-PCR, cDNA fragments covering appropriate intron/exon boundaries (DPLPs: Exons 1–3; GAPDH: Exons 5–8) were cloned in series into pBluescriptII KS(+). Plasmids containing rat Kv4.2, human KChIP3a, and human KChIP4bL cDNAs were obtained as previously described [12], [17], [23]. The complete rat DPP6a (rDPP6a) cDNA was cloned from a rat cerebellar cDNA library using overlap extension PCR. Rat DPP6S cDNA was generated by amplifying the DPP6S 5′ sequence from a rat cortical cDNA library and swapped into the rDPP6a construct with the help of an internal BglII site. Rat DPP6K 5′ sequence was amplified from a rat cerebellar cDNA library and swapped into the rDPP6a construct, similar to DPP6S. The DPP6K N-terminal deletion (ΔN16) mutant was generated by amplifying a PCR fragment encoding the truncation and swapping it into the DPP6K wild-type construct. All clones were constructed with strong 5′ Kozak sequences. Our original rat DPP6 clones differed at a single position from the Ensembl rat genomic sequence at E305V in the extracellular domain of DPP6a. We have mutated this site to the consensus and verified that this difference has no measurable effect on any functional properties (data not shown). Sequences for all DNA constructs were verified by automated sequencing (DNA sequencing facility, Baylor College of Medicine). RNA transcripts for oocyte expression were synthesized from linearized DNAs using mMessage mMachine high-yield capped RNA transcription kit (Ambion).

### Brain mRNA Harvest, cDNA Synthesis, and Quantitative RT-PCR

Cerebellar samples (10 mg each) were collected from P21 rats, and total RNA was isolated using the RNAqueous-Micro kit (Ambion). Reverse transcription (RT) reactions were conducted with the Superscript III kit (Invitrogen) using random hexamers and following manufacturer's instructions.

Procedures for quantitative real-time PCR were adapted from Liss et al. (2001) [31]. The following forward (f) and reverse (r) primers were used for nested PCR.

External primers:

GAPDH (362 bp): (f) GTCTTCACCACCATGGAGA, (r) ATGACCTTGCCCACAGCCT

Kv4.2 (406 bp): (f) GACAGACAATGAGGATGTCA, (r) GTGGTAGATCCGACTGAAG

DPP10a (270 bp): (f) TGGTTTGTCTTGGAACTCTG, (r) CTCTAGTGACAGTCTTGTTTC

DPP10c (263 bp): (f) GAGGAAGTGTGAGCTCCGA, (r) CTCTAGTGACAGTCTTGTTTC

DPP10d (246 bp): (f) ACCCAGCAGGAACTTAGAG, (r) CTCTAGTGACAGTCTTGTTTC

DPP6a (239 bp): (f) AGTTTGCAAGGTAGAGGATC, (r) GAGACAGACTGGTATCTTCC

DPP6K (257 bp): (f) GTGCTCACCAGAACAATGGA, (r) GAGACAGACTGGTATCTTCC

DPP6L (353 bp): (f) GCTGTACCAAAGGTTCACC, (r) GAGACAGACTGGTATCTTCC

DPP6S (165 bp): (f) CAGGAAAATCTGTACAGCAG, (r) GAGACAGACTGGTATCTTCC

Internal primers:

GAPDH (221 bp): (f) CCAAAAGGGTCATCATCTC, (r) ATCCACAGTCTTCTGAGTG

Kv4.2 (309 bp): (f) ACACTCCGAGTCTTTCGAG, (r) GCTCAGAGAGCAGATAGAC

DPP10a (140 bp): (f) CCATCACATCAAGTGTCAGC, (r) GGATGACAGACATTGTGATG

DPP10c (176 bp): (f) GATGACAGCCATGAAGCAG, (r) GGATGACAGACATTGTGATG

DPP10d (110 bp): (f) CAGAGAGGAACTGGGAAGT, (r) GGATGACAGACATTGTGATG

DPP6a (143 bp): (f) GTCCAATAACGTCAGGTGTC, (r) GGATGACTGAGGTGACAATC

DPP6K (178 bp): (f) GTGAGGTTCAAGACTCCAAG, (r) GGATGACTGAGGTGACAATC

DPP6L (125 bp): (f) GAGCGACTGTGACGAGGAG, (r) GGATGACTGAGGTGACAATC

DPP6S (123 bp): (f) TCTGTACAGCAGCAGGATCA, (r) GGATGACTGAGGTGACAATC

qRT-PCR was conducted using IQ SYBR Green Supermix (Bio-Rad) on a PTC-200 thermal cycler with a chromo-4 detection system (Bio-Rad) as described previously [20].

### *In Situ* Hybridization

*In situ* hybridizations with [^35^S]-labeled riboprobes were conducted as previously described [20]. In short, mounted sections were treated with proteinase K for 10 min, acetylated with acetic anhydride for 10 min, dehydrated with ascending concentrations of ethanol, and air dried. The sections were incubated with the riboprobes for 16–20 hrs at 60°C in the hybridization solution. The sections were then treated with RNase for 35 min to remove nonspecifically bound probes and washed 4 times under high stringency with solutions of decreased salinity at room temperature. Afterwards, they were washed once with SSC at high temperature for 35 min. Graded ethanol was then used to dehydrate the sections, and the sections were dried and laid apposed to X-ray film for 3 days at 4°C.

### Heterologous Expression in *Xenopus* Oocytes and Two-electrode Voltage Clamp Recordings

*Xenopus laevis* frogs were anesthetized with 0.1% Tricane solution absorbed through the skin, and stage V–VI oocytes were surgically harvested and defolliculated by collagenase I treatment. Oocytes were injected with Kv4.2 cRNAs with or without auxiliary subunit cRNAs using a Nanoinjector (Drummond Scientific Company). Injected oocytes were incubated at 18°C for 1–2 days in standard ND96 solution (in mM: 96 NaCl, 2 KCl, 1.8 CaCl_2_, 1 MgCl_2_, and 5 HEPES, pH 7.4 adjusted with NaOH) supplemented with 5 mM Na-pyruvate and 5 µg/ml gentamycin.

The two-electrode voltage clamp technique was used to elicit whole-cell currents from injected oocytes. The microelectrodes had <1 MΩ tip resistance and were filled with 3 M KCl solutions. The voltage-clamp amplifier (Oocyte Clamp OC-725, Warner Instruments) was under the control of WinWCP software (John Dempster, University of Strathclyde, Glasgow, UK). The data were digitized and low-pass filtered (model 902, Frequency Devices) at various frequencies depending on the sampling rate. The capacitative transient and linear leak were subtracted off-line by scaling up transients at voltages without ionic currents (at −70 mV) and subtracting them from total currents. Recordings with offsets >3 mV were removed from data analysis, and the average leak current was <0.2 µA.

### Patch-clamp Electrophysiology

CG cells were visualized using a fixed-stage upright microscope (BX51WI, Olympus) equipped with dedicated water-immersion objectives designed with long working distances (LUMPlanFL, 60x/0.90 W). Imaging of neurons was conducted using infrared (IR) and differential interference contrast (DIC) techniques. Patch pipettes were fabricated from thin-walled borosilicate glass capillaries (TW150-4, World Precision Instruments, Inc.) using a Flaming/Brown micropipette puller (model P-97, Sutter Instrument Co.) and had a resistance of 1–2 MΩ. An Axopatch 200B integrating patch clamp amplifier (Axon Instruments) was used to direct voltage pulses, and the data were acquired using pClamp 7 (Axon Instruments) program on a desktop personal computer.

Pipettes were fire polished and had areas near their tips wrapped with parafilm to reduce electrode capacitance. Tight-seal whole-cell recordings were obtained using standard techniques. The patch electrodes were backfilled with solution containing (in mM): 120 K-gluconate, 20 KCl, 5 NaCl, 10 HEPES, 4 Mg_2_-ATP, 0.3 Tris-guanosine 5′-triphosphate (GTP), 14 phosphocreatine, pH = 7.25 after adjustment with KOH; 301 mOsm). Series resistance and capacitance were determined by optimal cancellation of the capacitative transient, and the series resistance was typically 3–5 MΩ. If the series resistance changed more than 10% of the initial value during recording, the recording was terminated or the data was discarded. The current signals were recorded using an Axopatch 200B and filtered at 5 kHz and sampled at 10 kHz. The liquid junction potential between the electrode and bath solutions was calculated to be ∼−14.2 mV, and the data has been corrected for this voltage. All experiments were conducted at RT (22–23°C). Ohmic leak and capacitative currents were subtracted off-line using scaled-up versions of null traces at low voltages.

### Isolation of I_SA_

Non-I_SA_ currents were suppressed by modifications of the external bath solution and by pharmacological agents. The modified ACSF bath solution was low in Ca (in mM: 84 NaCl, 4 KCl, 0.1 CaCl_2_, 3.1 MgSO_4_, 1 NaH_2_PO_4_, 1 NaHCO_3_, 1 glucose; 300–320 mOsm) to reduce contamination by Ca currents and Ca-dependent K currents. Supplementation with 0.5 µM tetrodotoxin (TTX), 40 mM tetraethylammonium (TEA), and 100 µM 4-aminopyridine (4-AP) (Sigma-Aldrich) respectively block Na currents, non-inactivating K currents, and D-currents. TEA supplementation is accomplished by substituting TEA for equimolar amount of NaCl. In some experiments, 100 µM linopirdine (Sigma-Aldrich) and 100 µM ZD7288 (Tocris) were used to block M-current and H-current, respectively.

In addition, I_SA_ was reliably isolated using a subtraction protocol that exploits the difference between the high- and low-threshold K currents. Currents were recorded in response to depolarizing steps to various voltages from a conditioning step at either −114 mV or −44 mV for 1 sec. The I_SA_ is inactivated by prepulse to −44 mV, and the subtraction of the current after the −44 mV conditioning pulse from the current after the −114 mV conditioning pulse reveals the transient outward current during the test pulse. Tail reversal measurements gave I_SA_ reversal potential at −80 mV. Calculated E_K_ is −88.3 mV.

### Data Analysis

Data were analyzed with WinWCP (John Dempster, University of Strathclyde, Glasgow, UK), Clampfit 6 (Axon Instruments), and Origin softwares (OriginLab Corp.). Peak conductance (G_p_) was calculated as G_p_ = I_p_/(V_c_−V_rev_), where I_p_ is the peak current, V_c_ is the command voltage, and V_rev_ is the reversal potential (−90 mV in ND96). The G_p_-V curves were described using the first-order Boltzmann function: G_p_/G_p,max_ = 1/(1+exp(V_m_−V_0.5a_)/S_a_)), where G_p_/G_max_ is the fraction of maximal conductance, V_m_ is the membrane potential, V_a_ is the potential for half-maximal activation, and S_a_ is the slope factor. Steady-state inactivation was also described assuming a simple Boltzmann distribution, with the corresponding parameters of I/I_max_, V_0.5i_, and S_i_. Using Clampfit, the time courses of inactivation were fitted by a sum of two exponential terms, and the time courses of recovery from inactivation were described using a single exponential term initially and two exponential terms when the fitted curve deviated from experimental data significantly.

The number of DPP6 subunits per channel was examined by measuring the slowing of DPP6a-mediated fast inactivation via DPP6a∶DPP6K mixing and comparing it to the predicted inactivation slowing when only one DPP6a subunit is present. DPP6a-mediated fast inactivation is markedly faster than DPP6K-mediated inactivation; therefore, significantly reducing the DPP6a contribution to the mix should discernibly slow fast inactivation. Calculations for the predicted maximum amount of slowing in fast inactivation assume the use of simple single-step inactivation models for both DPP6a- and DPP6K-mediated inactivation:
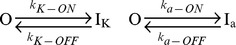
where O, I_a_, and I_K_ are open, inactivated (DPP6a), and inactivated (DPP6K) states, and *k_ON_* and *k_OFF_* are rate constants for inactivation and recovery from inactivation proceeding to and from the indicated inactivated states. The *k_ON_* and *k_OFF_* can be calculated from the following equations:
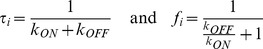
where τ_i_ is the time constant for fast inactivation and *f_i_* is the fraction inactivated by the fast mechanism. Kv4.2+KChIP3a channel with only DPP6a has a τ_i_ of 6 ms with a *f_i_* of 0.71 (Table 1). When only DPP6K is used, τ_i_ has a value of 35 ms with a *f_i_* of 0.74 (Table 1).

**Table 1:** Biophysical Properties of Kv4 Channel Complexes with Various DPP6 Variants.

Property	Kv4.2+KChIP3a				
	+DPP6K	w/o DPP6	+DPP6a	+DPP6S	+DPP6K/ΔN16
**G_p_-V Relation, (n)**	(3)	(10)	(3)	(6)	(3)
V_0.5a_ (mV)	−20.1±0.8	−17.4±0.9	−26.5±3.3	−25.7±1.2*	−15.8±0.2
S_a_ (mV/e-fold)	18.9±0.2	20.4±0.8	17.9±1.0	19.8±1.0	18.2±0.5
**Steady-state Inactivation, (n)**	(5)	(25)	(5)	(5)	(3)
V_0.5i_ (mV)	−72.4±0.6	−61.2±0.5*	−63.3±0.5*	−64.9±1.3*	−60.2±3.3*
S_i_ (mV/e-fold)	3.7±0.1	3.6±0.1	4.3±0.1*	4.7±0.1*	3.4±0.0
**Inactivation at +40 mV, (n)**	(3)	(6)	(8)	(4)	(3)
τ-1 (ms)	35.0±1.1	42.2±0.8*	6.0±0.4*	39.4±5.1	70.1±8.0*
τ-2 (ms)	189±14	192±3	461±23*	177±8.0	156±1.6
W-1 (%)	74±1	24±2*	71±1	63±2*	40±10*
W-2 (%)	21±0	69±2*	22±1	30±1*	51±13
W-ss (%)	4±1	6±1	7±1*	7±2	9±3
**Recovery from Inactivation, (n)**	(5)	(9)	(4)	(4)	(5)
τ-1 (ms)	43±4.4	76.7±1.0	15.3±1.6$	24.4±4.1$	32.5±5.2$
τ-2 (ms)	265±22				
W-1 (%)	29±3				
W-2 (%)	72±3				

See “Methods” for descriptions of parameters measured. τ-n and W-n represent the corresponding time constants and relative weights, expressed as percentages, derived from exponential fittings of the 1-sec traces. W-ss represents the relative weight of the steady-state component.

#, recovery at −100 mV measured with a prepulse of 1 sec.

* p<0.05, relative to Kv4.2+KChIP3a+DPP6K.

$ p<0.05, relative to Kv4.2+KChIP3a.

Since DPP6 proteins are known to form dimers [32], models were tested for both 2 DPP6 subunits per channel and 4 DPP6 subunits per channel. Two types of kinetic models were generated: a full proportionate ON-rate model (Model #1) and a DPP6a-only proportionate ON-rate model (Model #2):
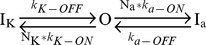
(Model \#1)

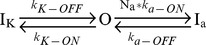
(Model \#2)Where N_a_ and N_K_ represent the number DPP6a and DPP6K present, respectively. For Model #1, the ON-rate for I_a_ or I_K_ is proportional to the respective number of DPP6a and DPP6K subunits present, and the OFF-rate is unchanged. For 4 DPP6's per channel, the predicted *k_a-ON_* and *k_a-OFF_* for DPP6a-mediated inactivation are respectively 30 s^−1^ per subunit and 48 s^−1^ per channel; for 2 DPP6's per channel, they are respectively 60 s^−1^ per subunit and 48 s^−1^ per channel. For 4 DPP6's per channel, the *k_K-ON_* and *k_K-OFF_* are respectively 5.8 s^−1^ per subunit and 7.5 s^−1^ per channel; for 2 DPP6's per channel, they are respectively 11.6 s^−1^ per subunit and 7.5 s^−1^ per channel. For Model #2, we assume that DPP6a specifically overlays an additional N-type inactivation process to a channel that would otherwise inactivate by a separate intrinsic inactivation process. In this case the intrinsic inactivation mechanism does not change much depending on DPP6 subunit composition (compare DPP6K and DPP6S kinetics Table 1 and see [22]), but the N-type inactivation process has an ON-rate that is proportional to the number of DPP6a subunits present. The intrinsic K_ON_ is 21 s^−1^ and the intrinsic K_OFF_ is 7.5 s^−1^. The DPP6a-specific kinetic rate constants are: for 4 DPP6's per channel, *k_a-ON_* is 25 s^−1^ per subunit and *k_a-OFF_* is 52 s^−1^ per channel; for 2 DPP6's per channel, *k_a-ON_* is 50 s^−1^ per subunit and *k_a-OFF_* is 52 s^−1^ per channel. Using the QuB software program (www.qub.buffalo.edu), we constructed the different models assuming only one DPP6a subunit per channel. The predicted value and amplitude for the fast inactivation time constant were calculated using the Equilibrium P function.

For modeling the effects of DPP6K on steady-state inactivation, we used a linear model based on an equal energetic contribution for each incorporated DPP6K subunit. Based on the magnitude of the shift produced by four DPP6K subunits, the model assumes a −2.3 mV shift in the steady-state inactivation curve for each DPP6K subunit incorporated in the channel. To confirm that this model gives a linear shift in the Boltzmann midpoint based on the DPP6K mole fraction, we constructed a set of Boltzmann curves based on the DPP6a curve with a −2.3×N_K_ mV shift in the DPP6a curve, where N_K_ is the number of DPP6K subunits in the mixed tetramer. These curves were then summed using the calculated mole fractions for the different subunit compositions based on the expression mix used and then the summed data was fit with a first-order Boltzmann distribution. For experimental averages, data are presented as mean ± standard error of the mean (SEM). Statistical significance was determined by comparing data sets using Student's two-tailed (independent) *t*-test and the significance level of p<0.05.

## Results

### Detection and quantitation of DPP6 transcripts in the cerebellum of p12 rats

**Figure 1:**
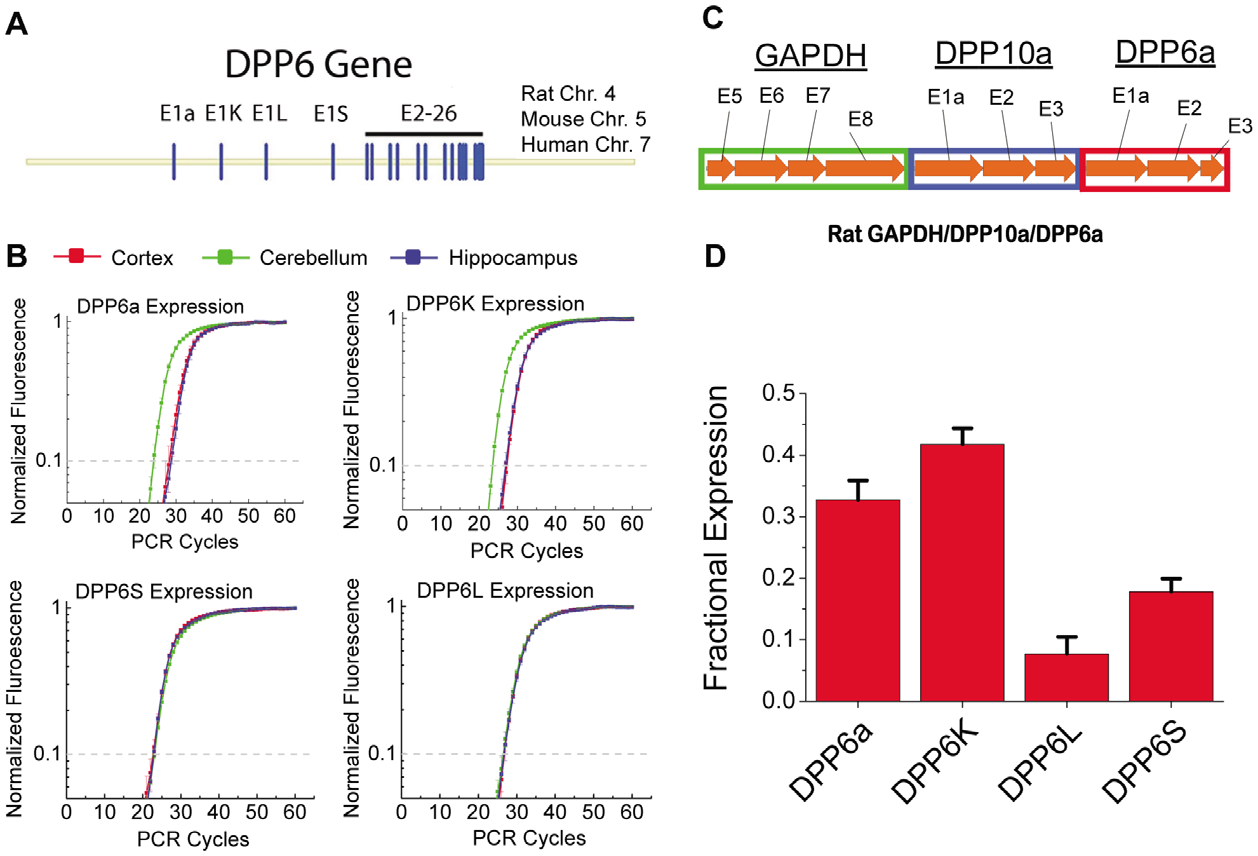
Analysis of DPP6 splice variant expression in CG cells. A) DPP6 gene shows a conserved set of four alternative first exons producing the protein variants DPP6a, DPP6K, DPP6L and DPP6S. B) qRT-PCR using SYBR Green Fluorescence for DPP6 variants from three brain regions: cortex, cerebellum and hippocampus. DPP6a and DPP6K show enhanced expression in cerebellum. C) Normalization controls used to correct for primer amplification efficiency differences. Amplification targets from GAPDH and two DPLP variants were diluted and used to construct amplification curves. Common GAPDH signal was used to ensure consistent dilution of standards. D) Relative expression levels of DPP6 variants in cerebellum following normalization. Due to high levels of expression in CG cells and the abundance of these neurons, these signals essentially report the relative expression of DPP6 variants in CG cells.

To determine the relative expression of different DPP6 variants in CG cells, we quantified the mRNA expression levels for DPP6 variants using qRT-PCR. PCR primers were designed to amplify variant-specific DNA sequences for each of the four DPP6 N-terminal variants expressed in rat brain (Fig. 1A). Forward primers specific for the different DPP6 Exon 1's were paired with common reverse primers in Exons 2 and 3 (see Methods). Amplification curves from the cortex, cerebellum, and hippocampus showed that DPP6K and DPP6a are expressed at higher levels in cerebellum, whereas DPP6S and DPP6L are expressed at similar levels in these three brain regions (Fig. 1B). While these results showed a clear difference in the regional expression patterns for different DPP6 variants, they could not be directly compared across variants without precisely knowing the efficiencies of the different primer sets.

Therefore, we constructed a set of standardizing DNA constructs to precisely analyze the amplification efficiency of different primer sets. Each of the constructs consists of Exon 5–8 of GAPDH, followed in tandem by Exons 1–3 of two DPLP variants. For example, one construct contains the Exons of GAPDH, DPP10a, and DPP6a (Fig. 1C). These constructs allowed us to control for amplification variability and directly tie the amounts of multiple different transcripts to a common GAPDH control, thereby maximizing the accuracy of our measurements. Following normalization of our amplification reactions using a series of dilutions for these standardizing constructs, we determined the relative expression levels for DPP6 N-terminal variants in cerebellum (Fig. 1D). Given that previous studies have demonstrated that DPP6 expression in cerebellum is predominately in CG cells [18], [19], these results reveal the average expression levels for DPP6 variants in CG cells. The results showed that the dominant transcript in CG cells is actually the DPP6K variant, accounting for 41±3% (n = 12) of all DPP6 messages (Fig. 1D). It also showed that DPP6a transcript is also abundant at 33±3% (n = 12) of total DPP6 transcripts, as predicted by our *in situ* hybridization results (Fig. S1) and a previous study [26]. It further indicated that DPP6S comprises only 18±2% (n = 12) of total DPP6 transcripts in CG cells and the DPP6L variant accounts for the remaining transcripts (8±3%, n = 12).

### DPP6K, the most common DPP6 variant expressed in CG cells, distinctly slows down recovery from inactivation of Kv4.2+KChIP3a channels

**Figure 2:**
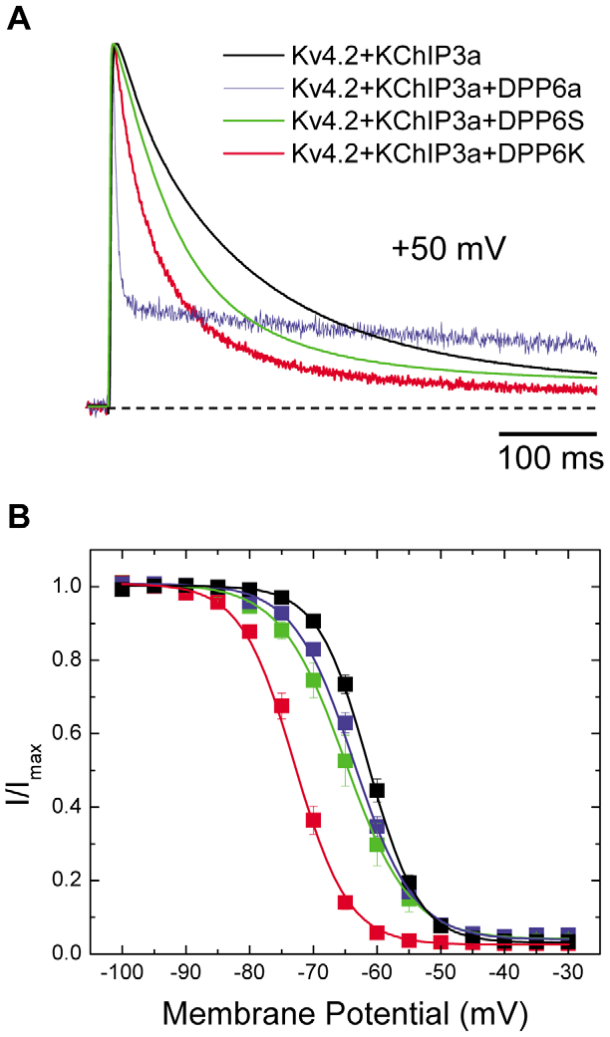
DPP6K dramatically accelerates inactivation kinetics and leftward shifts steady-state inactivation curve. *Xenopus* oocytes were injected with cRNAs encoding Kv4.2 and KChIP3a along with either DPP6a, DPP6S, or DPP6K. Transient currents were recorded using two-electrode voltage clamp. (A) Representative normalized current traces generated by voltage steps to +50 mV from a holding potential of −100 mV for 1 sec. Only the first 500 ms are shown. (B) Voltage dependence of steady-state inactivation for the various channel complexes. The steady-state inactivation protocol consisted of a 10-sec prepulse at the indicated potentials and a 250-ms test pulse to +50 mV, with an inter-episode interval of 5 secs. The fraction of available current (I/I_max_) was plotted against the prepulse membrane potential. Data are shown as mean ± SEM, and the lines represent fits using Boltzmann functions.

The high level of DPP6K in CG cells raised the prospect that this variant plays a special role in shaping I_SA_ properties in CG cells. A previous study of DPP6K did not identify any significant unique functional effect when co-expressed with Kv4.2 [19]; however, since the native I_SA_ channels also contain KChIP proteins, we sought to re-examine this question in a native-like ternary complex by combining DPP6K with Kv4.2 and KChIP3a, which are also highly expressed in CG cells [33], [34], [35], [36]. Following co-expression of DPP6K with Kv4.2 and KChIP3a in *Xenopus* oocytes the DPP6K-containing channels were found to have significant functional differences from channels expressed with other DPP6 variants. Similar to other DPP6 variants, activation and inactivation gating kinetics in the presence of DPP6K are accelerated compared to channels lacking DPP6; however, the extent of acceleration of inactivation is dependent on the variant being tested (Fig. 2A). To quantify the observed changes, the development of macroscopic inactivation at +40 mV in ternary complex channels containing DPP6a, DPP6S, or DPP6K were all adequately described by using the sum of two exponential terms (Table 1). Inactivation in the presence of DPP6K is not as fast as DPP6a, which has at its N-terminus an inactivation peptide that confers a distinctive ultra-fast, N-type inactivation with a τ of ≃6 ms [22]. However, DPP6K-associated channels inactivate significantly more rapidly than channels expressed with the DPP6S variant (Fig. 2A). Our analysis showed that the faster inactivation associated with DPP6K results not from changes in the time constants of the two components but rather a significant increase in the contribution from the fast inactivating component with a concomitant decrease in the contribution from the slow inactivating component (Table 1). The steady-state inactivation properties measured with DPP6K were also remarkably different from channels formed from other subunit combinations (Fig. 2B). The voltage of half-inactivation for Kv4.2+KChIP3a+DPP6K channels is consistently more hyperpolarized than Kv4.2+KChIP3a channels, Kv4.2+KChIP3a+DPP6S channels, or Kv4.2+KChIP3a+DPP6a channels (Table 1). In addition to a leftward shift in midpoint, the slope factor of steady-state inactivation for channels containing DPP6K is also significantly reduced than seen with DPP6S or DPP6a, but similar to channels lacking DPP6. These combined effects cause A-type channels containing DPP6K to favor the inactivated state at subthreshold membrane potentials compared to channels containing other DPP6 variants.

**Figure 3:**
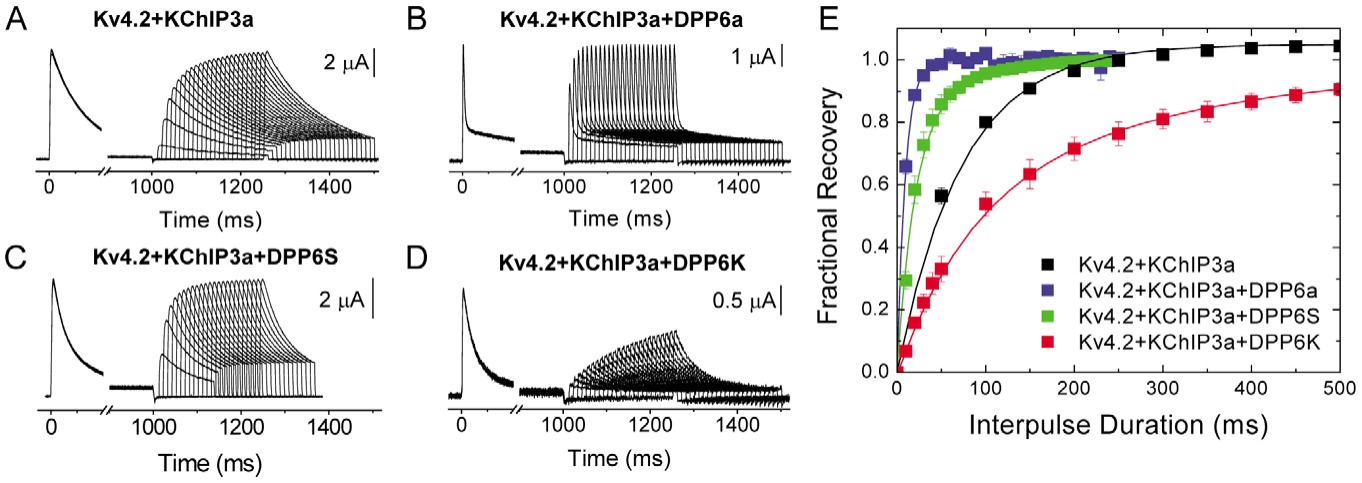
DPP6K markedly represses the recovery-accelerating effects of KChIP3a. Representative current traces generated during the two-pulse protocol used to measure recovery from inactivation for Kv4.2+KChIP3a (A), Kv4.2+KChIP3a+DPP6a (B), Kv4.2+KChIP3a+DPP6S (C), and Kv4.2+KChIP3a+DPP6K (D). A 1-sec depolarization to +50 mV was delivered to maximally inactivate the channels, followed by an increasing recovery interval at −100 mV before applying a 250-ms test pulse at +50 mV to check the degree of recovery from inactivation. (E) Fractional recovery was plotted as a function of the interval duration at −100 mV. The residual value at the end the first pulse was subtracted from the peak current values of the first and second pulses, and the fractional recovery was determined by dividing the peak value of the second pulse by that of the first pulse.

The stabilization of inactivation in DPP6K channels could be due to favoring entry into the inactivated state or slower recovery from inactivation, or both. To measure the effects of DPP6K on recovery from inactivation, we examined recovery from inactivation at −100 mV using a two-pulse protocol and plotted the fractional recovery as a function of the interpulse duration (Fig. 3A–3E). The length of the first pulse was set at 1 second to allow maximum amount of inactivation before testing for channel recovery. As shown in Figure 3A–3D, the addition of DPP6S or DPP6a dramatically accelerated the recovery kinetics of Kv4.2+KChIP3a channels. However, when DPP6K is co-expressed with Kv4.2+KChIP3a channels, the opposite effect occurs as the kinetics of recovery from inactivation are dramatically slowed (compare Fig. 3D with Fig. 3A; Fig. 3E). Furthermore, the time course requires the sum of two exponential functions for a proper description (Table 1). These results clearly show that, in the context of the native-like ternary complex channel, the highly-expressed DPP6K variant produces distinct modulation of channel function. Furthermore, they suggest that the DPP6K-specific effects are derived from its variable N-terminus.

### DPP6K unique functional effects relies on an evolutionarily conserved sequences within the variable N-terminal domain

**Figure 4:**
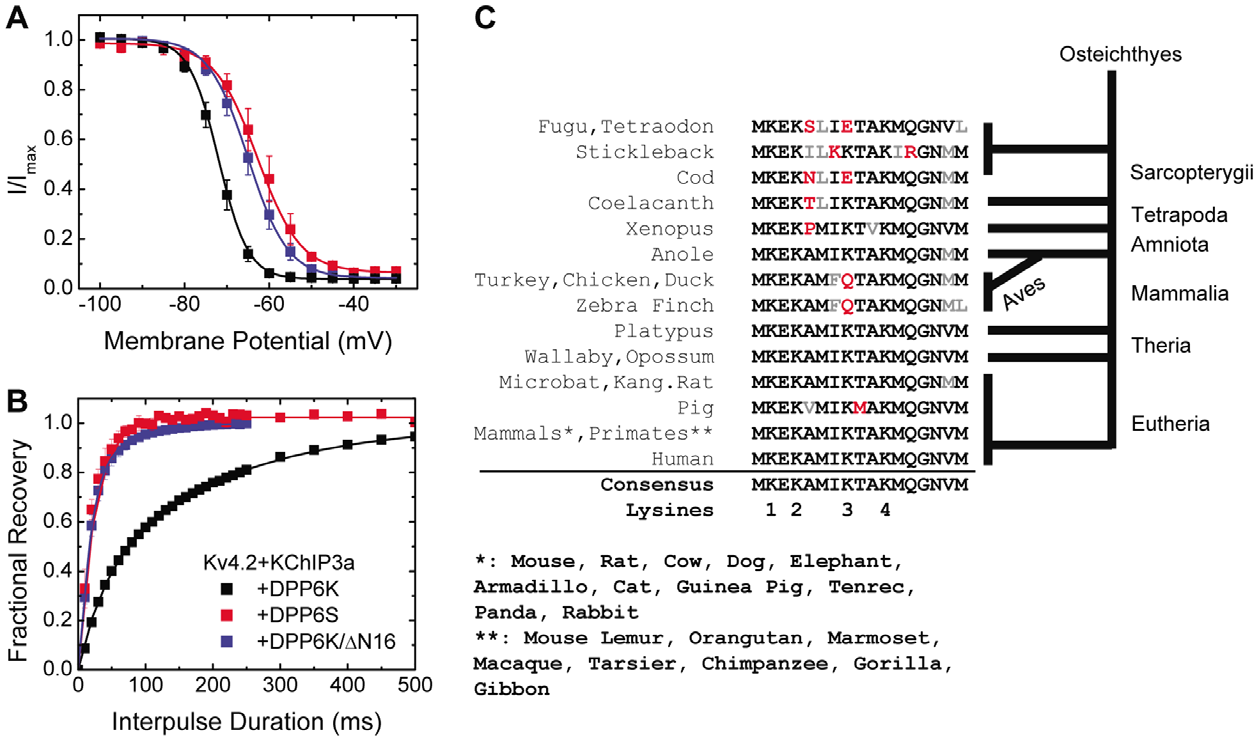
The DPP6K variable N-terminus is responsible for its distinct functional effects. (A) Voltage dependence of steady-state inactivation of ternary complex containing DPP6K and DPP6K/ΔN16 deletion mutant. The protocol was the same as that of Fig. 2B. (B) The kinetics of recovery from inactivation at −100 mV. The protocol was the same as that of Fig. 3. (C) Alignment of amino acid sequences of DPP6K variable N-terminus from various organisms. Consensus residues are shown in black; conservative substitutions, in gray; non-conservative substitutions, in red. Major evolutionary branch points indicated along the alignment relative to a terminus in placental mammals.

To determine whether it is the presence of the DPP6K N-terminus rather than the absence of DPP6a or DPP6S N-termini that is responsible for the DPP6K-differential effects, we deleted the DPP6K Exon 1K (E1K, Fig. 1A) sequence and generated the DPP6K mutant DPP6K/ΔN16 which initiates off of the native Met-17. Measurement of the steady-state inactivation of Kv4.2+KChIP3a channels co-expressed with wild-type DPP6K or with the DPP6K/ΔN16 deletion mutant showed that removing the unique N-terminus eliminates the DPP6K specific effects (Fig. 4A; Table 1). DPP6K/ΔN16 exhibits a large depolarization in the voltage of half-inactivation compared to DPP6K, with a final value similar to that of Kv4.2+KChIP3a+DPP6S channels. A parallel regulation of recovery from inactivation was also detected where slow recovery is lost when the variable N-terminus is deleted from DPP6K, resulting in recovery that is just as fast as ternary complex channels expressing DPP6S (Fig. 4B; Table 1). In conclusion, the highly conserved N-terminal domain of DPP6K is responsible for producing the unique functional effects of this specific variant.

Given that the DPP6K N-terminus is responsible for its unique functional effects, we sought to determine if it has been significantly conserved over evolutionary time. We examined DPP6 genes from a large number of species and found an obvious DPP6 Exon 1K sequence in vertebrate genomes from teleost and coelacanth fish to humans. The alignment of these sequences shows extremely high levels of sequence conservation over this long evolutionary time span with at most 2 non-homologous substitutions out of 17 positions for the most divergent teleost fish sequences (Fig. 4C). Indeed, by the appearance of mammals, the sequence found in humans was essentially fully fixed, with more than 85% of the mammalian genomes examined containing the unmodified consensus sequence. Preliminary screening of the incomplete elephant shark and lamprey genomic sequences did not yield Exon 1K homologs, although other DPP6 exon homologues could be found. Despite the incomplete nature of many primitive vertebrate genomes, we can say at least that DPP6 Exon 1K was fixed in the genome prior to the divergence of teleost fish around 380 million years ago. The extremely high conservation of DPP6K N-terminal domain supports an important conserved function for this domain and suggests its unique functional effects are likely highly conserved throughout the vertebrates.

### The functional effects of combining DPP6a and DPP6K variants

**Figure 5:**
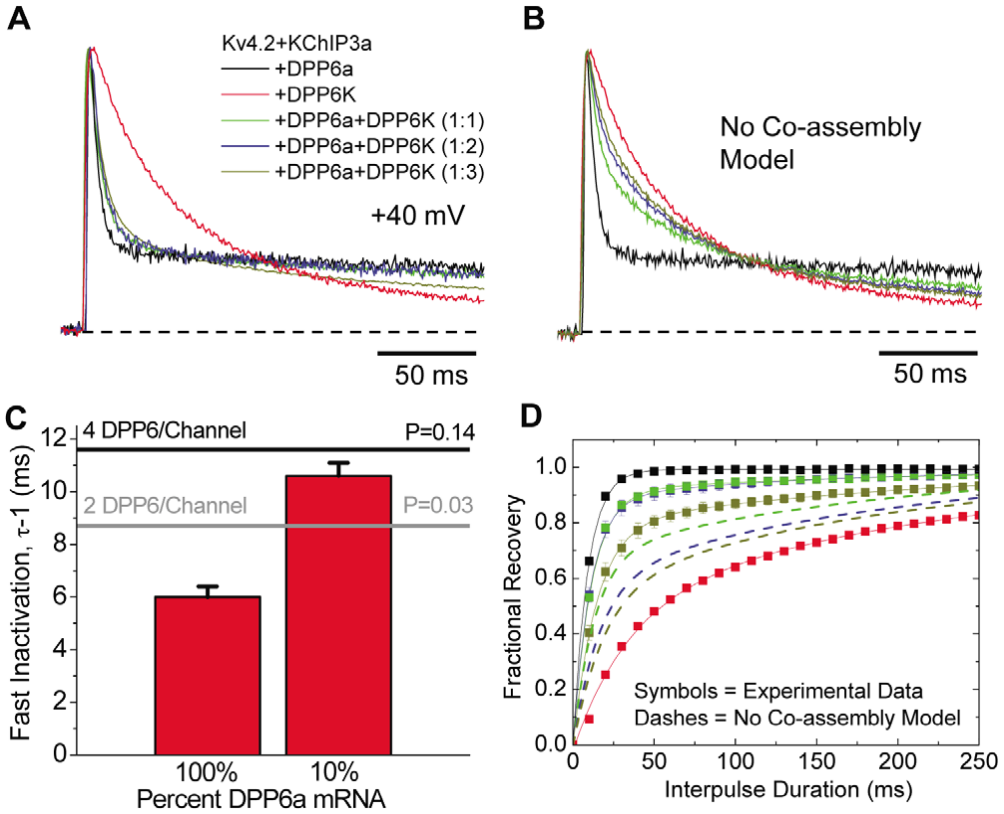
Co-assembly of DPP6K and DPP6a in heteromultimeric channel complexes. (A) Outward currents expressed by oocytes co-injected by various combinations of cRNAs, as elicited by depolarization to +40 mV from holding potential of −100 mV. (B) Expected rise and decay of currents if DPP6a and DPP6K subunits do not co-assemble and produce segregated channel populations containing either one alone. (C) Slowing of the time constant of fast inactivation when DPP6a mRNA changes from 100% to 10% mixed with DPP6K mRNA. To get the average value for fast inactivation, the slow phase of inactivation and non-inactivating current were described by exponential fitting and subtracted from the total current. The remaining average fast inactivation time constant was measured by taking the peak current for the fast inactivating fraction divided by its area. The average time constant measured by this method was very similar to the time constant measured by the best single exponential fit to the fast inactivating component. The black and gray lines show the predicted maximal slowing of fast inactivation with four DPP6 and two DPP6 per channel, respectively, with only 1 DPP6a subunit per channel. (D) Recovery from inactivation at −100 mV after a 200 ms-long prepulse (symbols) as compared to predicted results assuming no co-assembly of DPP6a and DPP6K (dashes).

Based on our qRT-PCR results, nearly 75% of all DPP6 transcripts in CG cells consist of either DPP6a or DPP6K, both of which separately confer distinct functional properties. An important question is: What are the functional consequences of forming channels with both DPP6a and DPPK in the same channel complex? Previous study has suggested that four DPP6 proteins may be co-assemble onto a Kv4-based channel, although DPP6 in solution tends to form dimers [30], [32]. Because of variant co-assembly, there are a large number of potential DPP6 subunit compositions that can exist on native I_SA_ channels. To begin to address these questions, DPP6a and DPP6K were co-expressed with Kv4.2 and KChIP3a at a series of different cRNA ratios (DPP6a∶DPP6K from 1∶1, 1∶2, and 1∶3). We first verified that the expression results are consistent with co-assembly of DPP6 variants on the channel. As Fig. 5A shows, even in channels expressed with a DPP6a∶DPP6K ratio of 1∶3, the rapid N-type inactivation produced by the DPP6a N-terminus is clearly evident in the current decay. This dominant effect of DPP6a would not occur if the DPP6 variants were not co-assembling and segregating onto different channels, resulting in channels that predominantly behave like DPP6K type channels (Fig. 5B). Although the DPP6a-mediated N-type inactivation is predominating, the kinetics for inactivation is clearly slowing as the fraction of DPP6K in the expression mix is increasing. As Table 2 shows, the time constant of fast component significantly increased from 6.0±0.4 ms (n = 8) for DPP6a-only channels to 8.5±0.1 ms (n = 5) for DPP6a∶DPP6K ratios of 1∶3 (P = 0.0003).

**Table 2:** Comparison between native I_SA_ from CG Cells and Reconstituted I_SA_ channels.

Property	CG cell I_SA_	Kv4.2+KChIP3a+			Kv4.2+KChIP4bL+		
DPP6a∶DPP6K ratio		(1∶1)	(1∶2)	(1∶3)	(1∶1)	(1∶2)	(1∶3)
**G_p_-V Relation, (n)**	(7)	(3)	(4)	(5)	(3)	(3)	(4)
V_0.5a_ (mV)	−25±3	−25.6±1.7	−25.6±1.7*	−22.9±0.9	−31.0±1.7	−30.9±0.1&	−34±1.5
S_a_ (mV/e-fold)	19±1.4	18.3±0.7	16.4±0.7*	17.2±0.4	12.4±0.2	12.1±0.1&	12.2±1.2
**Steady-state Inactivation, (n)**	(9)	(3)	(6)	(11)	(3)	(7)	(9)
V_0.5i_ (mV)	−76±1	−66.0±0.4	−68.8±0.7&	−69.6±0.5	−64.4±0.2	−70.6±0.7&	−68.4±0.8
S_i_ (mV/e-fold)	8.4±0.5	4.5±0.0	4.5±0.1&	4.5±0.1	4.5±0.2	4.5±0.2&	4.2±0.2
**Inactivation at +40 mV, (n)**	(6)	(3)	(3)	(5)	(3)	(3)	(4)
τ-1 (ms)	11±1	7.4±0.2	6.9±0.4&	8.5±0.1	6.9±0.1	5.1±0.2&	5.5±0.2
τ-2 (ms)	120±16	131±6	107±3*	122±3	90±4	67±3&	63±2
W-1 (%)	72±2	69±0	70±2*	65±1	62±1	71±1&	70±0
W-2 (%)	25±3	15±1	16±3&	25±1	25±1	16±1&	17±1
W-ss (%)	3±1	16±1	13±1&	10±0	12±2	12±0&	12±1
**Recovery from Inactivation** # **, (n)**	(3)	(3)	(3)	(6)	(3)	(3)	(3)
τ-1 (ms)	8±1	11.0±0.1	10.6±0.4&	14.5±0.9	11.3±0.9	14.3±0.9&	12.7±0.5
τ-2 (ms)	68±25	199±1.7	156±26&	207±15	111±22	145±26&	159±36
W-1 (%)	80±7	91±1	88±3*	79±2	76±3	63±3&	78±1
W-2 (%)	20±7	9±1	12±3*	21±2	24±3	37±3&	22±1

See “Methods” for descriptions of parameters measured. τ-n and W-n represent the corresponding time constants and relative weights, expressed as percentages, derived from exponential fittings of 200 ms-long pulse of both native and reconstituted currents. W-ss represents the relative weight of the steady-state component.

# , recovery at −100 mV measured with a prepulse of 200 ms.

* p>0.05.

& p<0.05.

P-values were determined relative to CG cells.

To further investigate whether DPP6a and DPP6K co-assemble as four- or two-DPP6 subunits per Kv4.2 channel complex, simple kinetic models were developed to determine how much slowing of the fast inactivation time constant would occur if DPP6a expression was dramatically reduced in our DPP6a∶DPP6K co-expression studies. We showed previously that DPP6a inactivates by an N-type mechanism [22], and studies show that in N-type inactivation, the inactivation ON-rate is proportional to the number of inactivation particle present [37]. The inactivation process with DPP6K is less well understood but appears to engage Kv4 intrinsic, non-N-type mechanisms [6]. Therefore, DPP6K inactivation was modeled in two ways: 1) Full proportional ON-rate model (Model #1), where DPP6K inactivation behaves like DPP6a in a way that the inactivation ON-rate is proportional to the number of DPP6K subunits incorporated, and 2) DPP6a-only proportional ON-rate model (Model #2), where the inactivation kinetics seen with DPP6K are independent from the DPP6 subunit composition (See Methods). We then calculated the predicted kinetics of fast inactivation when there is only one DPP6a subunit per channel for both two DPP6 and four DPP6 per subunit models and compared the prediction to the fast inactivation time constant measured when DPP6a and DPP6K cRNAs were co-injected at a ratio of 1∶9 along with Kv4.2 and KChIP3a cRNAs.

With a 1∶9 ratio of DPP6a∶DPP6K, the resulting current exhibited a peak conductance-voltage relationship with a midpoint of −16.1±2.8 mV and slope factor of 20±1.1 mV/e-fold (n = 3). With the large excess of DPP6K, the time constant of the fast phase of inactivation was slowed to 10.8±0.5 ms (n = 4). With only one DPP6a subunit, Model #1 predicts a fast time constant of 11.6 ms for a four DPP6 per channel model and 8.7 ms for a two DPP6 per channel model. Model #2 predicts a fast time constant of 11.6 ms and 8.75 ms for the four DPP6 per channel and two DPP6 per channel with only one DPP6a subunit assembled. The observed slowing is significantly greater than the maximum amount of slowing predicted for both two DPP6 per channel models (P = 0.03). Compared to the four DPP6 per channel models, the measured time constant is slightly less than the predicted maximum but not significantly different (P = 0.14). Given that in this sort of mixing experiment some channels will have multiple DPP6a subunits, we would expect our measured fast time constant to be slightly less than the predicted 11.6 ms. In addition, we find that the amount of inactivation occurring by the fast N-type mechanism with 10% DPP6a cRNA is greater than predicted, with the observed relative weight of ∼46% significantly greater than the predicted values of 14% for heterodimer and 24% for heterotetramer. This suggests that channels with DPP6a are over-represented in our recordings by a factor of about two. Nevertheless, our data supports the model that there are most likely four DPP6 subunits per channel since this overrepresentation of DPP6a would only make the measured time constant additionally faster than predicted.

To complement our analysis of inactivation kinetics, an examination of the effect of mixing DPP6a and DPP6K on the kinetics of recovery from inactivation also clearly showed the effects of heteromultimeric assembly of DPP6 variants into the Kv4 channel complex. Kinetics for recovery from inactivation with mixed expression of DPP6a and DPP6K are dominated by the rapid recovery from DPP6a induced N-type inactivation (Fig. 5D). If DPP6a and DPP6K subunits were not co-assembling on the same channel, recovery would be dominated by DPP6K (Fig. 5D).

**Figure 6:**
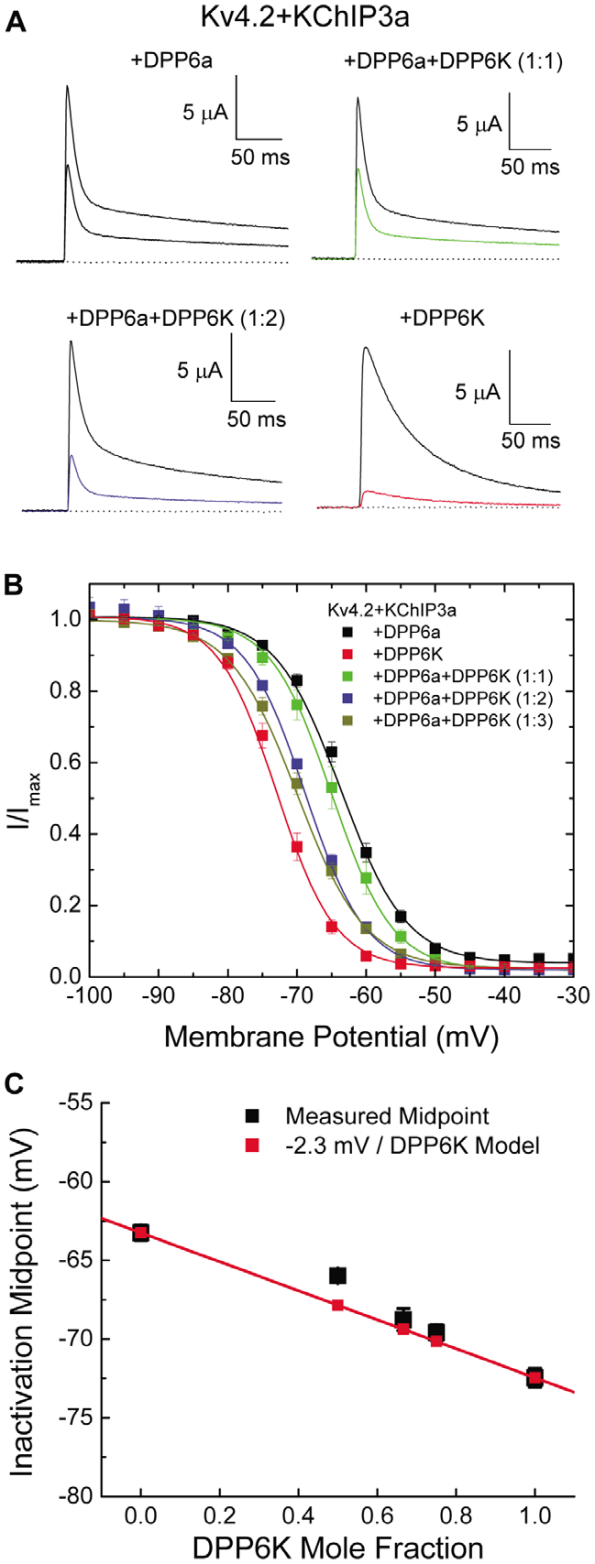
Steady-state inactivation of DPP6a∶DPP6K mixed channels. (A) Representative traces for Kv4.2+KChIP3a channels co-expressed with DPP6a alone, DPP6K alone, or with a DPP6a∶DPP6K mixture at 1∶1 or 1∶2 ratios, showing changes in steady-state inactivation at −65 mV. For the colored traces, the channels were held for 30 sec at −65 mV before pulsing to +40 mV for 250 ms to test available current. The black traces show the total currents, from test pulses where the channels were held at −100 mV and experienced no inactivation. (B) Voltage dependence of steady-state inactivation of ternary complexes with homotetrameric and heterotetrameric DPP6 subunits. (C) Progressive shifting of inactivation midpoint with increasing DPP6K ratio. The V_0.5i_ values were plotted against the calculated DPP6K mole fraction. Model assumes independent energetic effects for each DPP6K subunit incorporated into the channel, with symbols measured V_0.5i_ values for summed Boltzmann curves from the model.

The most consistent impact of DPP6K is on the steady-state inactivation properties for the channel. If we compare the maximum current in response to a depolarization to +40 mV, we see that holding at −65 mV reduces the amplitude of current evoked from DPP6a containing channel in half. As the ratio of DPP6K is increased, this steady-state inactivation at −65 mV increases until only about 10% current can be evoked with DPP6K alone (Fig. 6A). The steady-state inactivation curves produced by these mixtures show the progressive leftward shift in midpoint as DPP6K expression is increased, with no discerning change in slope factor (Fig. 6B; Table 2). When DPP6a-to-DPP6K ratio reached 1∶9, the midpoint and slope factor of steady-state inactivation were not significantly different from that of channel complexes with DPP6K alone (Kv4.2+KChIP3a+DPP6a+DPP6K (1∶9): V_0.5i_ = −73.3±0.3 mV; S_i_ = 4.1±0.2 mV/e-fold, n = 3). If we plot the inactivation midpoint versus the mole ratio of DPP6K we see that there is a progressive shift in midpoint as the ratio of DPP6K increases. Linear regression analysis shows a strong correlation consistent with the model that DPP6 subunit incorporation produces a consistent additive energetic shift in the inactivation midpoint of −2.3 mV for each DPP6K subunit incorporated into the channel (Fig. 6C). Our results suggest that the DPP6K expression ratio may be an important parameter neurons can use to tune the location of the I_SA_ steady-state inactivation curve relative to the neuron's resting membrane potential.

### Comparing the functional properties of native I_SA_ from CG cells with those of reconstituted A-currents heterologously expressed in *Xenopus* oocytes

To better understand the role that co-assembly of DPP6a and DPP6K plays in shaping the native I_SA_ from CG cells, we compared heterologously expressed currents in *Xenopus* oocytes to the native CG cell I_SA_. Previous recordings of CG cells from Kv4.2 knock-out animals have suggested that Kv4.2 is the most important α-subunit, required for 80% of the CG cell I_SA_ [28]. *In situ* hybridization results have also shown that CG cells express primarily KChIP3 and KChIP4 [34], [35]. Moreover, published RT-PCR results have demonstrated that KChIP3a is the predominant KChIP3 variant in CG cells, where all four KChIP4 variants are expressed similarly. Based on these observations, we decided to test how well channel complexes composed of Kv4.2 and either KChIP3a or KChIP4bL (long variant) co-expressed with DPP6a and DPP6K at reasonable expression ratios recapitulate the functional properties of I_SA_ channels in CG cells.

**Figure 7:**
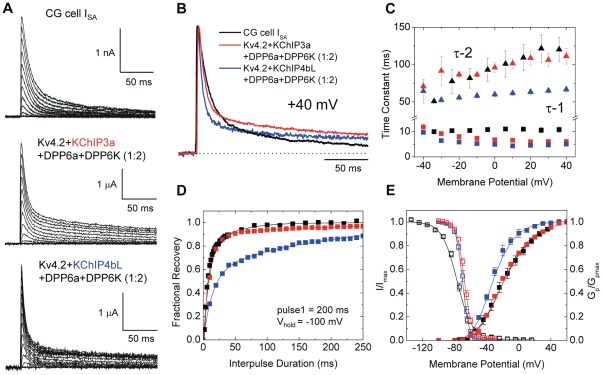
Reconstitution of native I_SA_ channel from CG cells by heterologous expression in oocytes. (A) Outward transient currents elicited from CG cells and oocytes expressing Kv4.2, a mixture of DPP6a and DPP6K at 1∶2 ratio, and either KChIP3a or KChIP4bL. From a holding potential of −100 mV, either a 200-ms (CG cells) or 1-sec (oocytes) step depolarizations were made from −100 mV to +40 mV at 10 mV increments. (B) Overlapped normalized current traces at +40 mV from the indicated channels. (C) Time constants of inactivation at indicated membrane potentials for I_SA_ from CG cells, Kv4.2+KChIP3a+DPP6a+DPP6K (1∶2), and Kv4.2+KChIP4bL+DPP6a+DPP6K (1∶2). (D) Recovery from inactivation at −100 mV, measured using the two-pulse protocol. (E) Normalized peak conductance-voltage relations (G_p_/G_p,max_) and steady-state inactivation curves (I/I_max_) for I_SA_ from CG cells and reconstituted channel complexes.

In recordings using whole-cell patch clamp from acute cerebellar slices, we found that the native I_SA_ of CG cells rises rapidly and decays with a clearly evident two exponential time course, in agreement with previous reports [38], [39]. As the membrane potential becomes more positive, the rapid inactivating component (τ-1≃11 ms at +40 mV) becomes more prominent than the slow inactivating phase (τ-2≃120 ms at +40 mV) (Fig. 7A). As for the voltage dependence of the two inactivating components, the fast phase of inactivation is voltage-independent above −10 mV, but for the slow phase, inactivation becomes progressively slower with increasing depolarization (Fig. 7C). This inverse relationship between voltage and inactivation kinetics has been described in native I_SA_ of CG cells by analyzing time constants of exponential fittings or half-inactivation times [29], [39].

When Kv4.2 is co-expressed in oocytes with either KChIP3a or KChIP4bL and with DPP6a and DPP6K at a 1∶2 ratio, implementation of voltage protocols similar to the one used for CG cells elicited outward currents that activated and inactivated rapidly (Fig. 7A). The currents decayed with a two exponential time course, similar to native I_SA_. In general, the shape of reconstituted currents and their measured time constants are similar to those of native I_SA_ (Fig. 7B & 7C). The primary contributors of this similarity between the native and reconstituted currents are the relative weight (W) of the fast component and the time constant (τ) of the slow inactivating component (Table 2). Neither parameter is statistically different from one another. However, the reconstituted channels inactivated significantly more quickly than the native I_SA_ during the fast phase and possessed a greater amount of residual current at the end of the 200 ms pulse (Fig. 7A; Table 2). To test whether the inactivation accelerating effects specific to DPP6K is an important contributing factor to CG cell I_SA_ waveform, DPP6S was substituted for DPP6K in co-expression studies. When Kv4.2 and KChIP3a were co-expressed with DPP6a and DPP6S at a 1∶2 ratio, the expressed current inactivated with a fast component with τ-1 of 7.5±0.03 ms and W-1 of 0.65±0.01 (n = 3). Notably, inactivation of the slow component exhibits a τ-2 of 349±9 ms and W-2 of 0.22±0.0 (n = 3). The significant difference between the τ-2 of native I_SA_ and ternary channels with DPP6S suggests that it is DPP6K that importantly contributes the slow phase of I_SA_ inactivation.

We next compared native I_SA_ and reconstituted channels composed of Kv4.2, KChIP3a, and a mixture of DPP6a and DPP6K at a 1∶2 ratio for their kinetics of recovery from inactivation. As Fig. 7D shows, the kinetics of recovery from inactivation for reconstituted current closely matches that of the native I_SA_. Both currents recover bi-exponentially with similar kinetics and relative weights for the two components (Table 2). Since our study has shown that for the reconstituted channel the slow phase of recovery is contributed by the incorporation of DPP6K, this result suggests that DPP6K in the native current also mediates the slow phase of recovery.

For voltage-dependent gating parameters, we examined the peak conductance-voltage (G_p_-V) relationship and the voltage dependence of steady-state inactivation (Fig. 7E; Table 2). The G_p_-V relationship of the reconstituted channels matches that of native I_SA_ channel with similar activation midpoint and slope factor. However, compared to native I_SA_ channel, the reconstituted channel shows steady-state inactivation that has a significantly depolarized midpoint and reduced slope factor. This suggests that steady-state inactivation property is being differentially modulated by unknown factors between CG cells and *Xenopus* oocytes.

Finally, we also examined the possibility that the native I_SA_ channel may contain KChIP4 auxiliary subunits rather than KChIP3a. Therefore, KChIP4bL was co-expressed in oocytes with Kv4.2 along with DPP6a and DPP6K at 1∶2 ratio. Overall, the results showed that ternary complex channels formed with KChIP4bL are more different from the native I_SA_ channels than those expressed with KChIP3a, but the exact kinetic and steady-state gating properties are sufficiently different from CG cell I_SA_ to suggest that KChIP3a is the more likely auxiliary subunit for most native channels. In Figures 7B and 7C, we show that the inactivation kinetics of both the fast and slow phases with KChIP4bL are considerably faster than the native I_SA_ or those produced with KChIP3a (Fig. 7C; Table 2). We also find that recovery is much slower with KChIP4bL than the native CG cell I_SA_ or with KChIP3a co-expression because of a much larger component of slow recovery (Fig. 7D; Table 2). Finally, the G_p_-V relationship for Kv4.2+KChIP4bL+DPP6a+DPP6K (1∶2) channels does not match that of native I_SA_, with the half-activation voltage shifted to the left by greater than 10 mV compared to I_SA_ from CG cells or channels co-expressed with KChIP3a (Fig. 7E; Table 2). In summary, we find that the specific KChIP variant expressed has important interactions within the ternary channel complex to shape the functional properties of the channel.

## Discussion

We have determined the expression levels of DPP6 N-terminal variants in CG cells and examined their potential impact on the properties of native I_SA_. As a result, it was discovered that DPP6K is the most common DPP6 variant in these neurons, and that the DPP6K variable N-terminal domain has evolved to markedly slow down recovery from inactivation and leftward shift the steady-state inactivation of I_SA_. In terms of channel recovery, DPP6K appears to act as a competitive inhibitor of the accelerating effects of KChIPs and DPLPs on recovery from inactivation. Kv4.2+KChIP3a channels co-expressed with DPP6K recover more slowly than those with DPP6S or DPP6a, and moreover, they recover more slowly than a binary complex of Kv4.2+KChIP3a. However, DPP6K's effects are limited by the co-expression of DPP6a: there is less pronounced slowing of recovery from inactivation and a less pronounced shift in the steady-state inactivation curve. On the other hand, DPP6K still significantly accelerates a slow phase of inactivation as well as increases a slow component of recovery from I_SA_ inactivation. By comparing reconstituted channels in heterologous expression systems with those of native I_SA_ channels, our results suggest that the subunit composition of I_SA_ channels is carefully controlled in order to finely tune the properties of neuronal I_SA_.

### DPP6K, another DPP6 N-terminal variant with unusual functional domain

Previously, we demonstrated that the DPP6a variant, along with its paralog DPP10a, utilizes its N-terminus encoded in Exon 1a to confer ultra-fast inactivation via a pore-blocking N-type mechanism [22]. Even though it has been reported that N-type “ball-and-chain” inactivation is produced by auxiliary subunits of other channels, such as Kvbeta of Kv1 channels and beta subunits of BK potassium channels [40], [41], [42], this was the first time that N-type inactivation was identified on a Kv4 channel auxiliary subunit and established that the variable N-terminal domain of DPLPs can confer unique functional properties to channels.

In this paper we discovered that the N-terminal domain of DPP6K also confers unexpected unique functional properties. In the initial paper describing DPP6K, Nadal and colleagues (2006) described only moderate effects on Kv4.2 channels associated with the DPP6K variant that were not affected by truncation of the entire variable DPP6K N-terminus [19]. We have confirmed similar modest effects by DPP6K on Kv4.2 channels in the binary channel complex (data not shown), but our results showed that in the ternary Kv4-KChIP-DPLP complex, DPP6K produces dramatic effects on recovery from inactivation. A possible reconciliation of these different results is to note that KChIPs accelerate the recovery of Kv4 channels from inactivation. Therefore, our Kv4.2+KChIP3a+DPP6K results are consistent with the hypothesis that the DPP6K N-terminus disrupts KChIP effects on recovery. Importantly, these results clearly demonstrate again that the variable N-termini of DPP6 and DPP10 can encode important modulatory domains that significantly influence the functional properties of the Kv4 ternary complexes.

In addition to slowing recovery from inactivation of Kv4.2+KChIP3a channels, DPP6K is also associated with a large hyperpolarizing shift in steady-state inactivation that is greater than those produced by DPP6S or DPP6a. Interestingly, the ΔN16 truncation that eliminates the DPP6K-mediated slowing of recovery also removes the hyperpolarizing shift in steady-state inactivation. This co-regulation by the DPP6K N-terminus is reasonable since slowing the rate of recovery from inactivation will favor the inactivated state and leftward shift the steady-state inactivation curve, assuming that the rate of low-voltage inactivation does not change. Further experiments will be required to elucidate the exact molecular mechanism by which DPP6K's modulatory domain disrupts recovery from inactivation and thereby hyperpolarizes the steady-state inactivation.

### DPP6a limits the DPP6K effects when the two variants are co-expressed

Our results showed that DPP6K and DPP6a are the two main DPP6 variants expressed in CG cells, with DPP6a constituting about 33% of total DPP6 transcripts. The finding that DPP6K is the most highly expressed DPP6 variant in cerebellum appears consistent with previous reports that show, among the four DPP6 variants expressed in CG cells, only DPP6K transcripts markedly exhibit a robust rostral-to-caudal gradient of decreasing level of expression [19], and this gradient is observed prominently at the total DPP6 protein level [24]. Since Kv4.2 also displays the same gradient [24], it is likely that DPP6K is an particular important component of the I_SA_ channel complex.

The reconstitution of I_SA_ from CG cells in oocytes showed that in ternary Kv4 channel complexes when DPP6a is present, the DPP6a-mediated N-type inactivation dominates. The strength of N-type inactivation over overall inactivation kinetics was also tested previously when Kv4.2 and DPP10a were co-expressed with KChIP4a, a KChIP4 variant that dramatically suppresses inactivation and KChIP core-mediated acceleration of recovery from inactivation [20]. It is consistent with the notion that the pore-blocking inactivation mechanism used by DPP6a and DPP10a may requires a single blocking particle and thus dominates slower forms of inactivation that involve conformational changes of the Kv4 pore-forming domains. As a result, the dominant fast phase of recovery from inactivation of channels co-expressed with DPP6a or DPP10a likely corresponds to the rapid release of the ball domain during hyperpolarization [22]. In the presence of DPP6a, DPP6K has little to no effects on recovery following short depolarizations; therefore, it seems clear that DPP6K effects on recovery are limited to inactivation caused by subunit conformational changes rather than pore block.

Consequently, DPP6K controls the slower inactivation and slower recovery from inactivation processes in a manner consistent with the native properties of the native I_SA_ from CG cells. Because the other DPP6 variants do not produce similar effects on slow inactivation and slow recovery (data not shown), these results suggest that DPP6K has a critical role in shaping the time- and voltage-dependent inactivation of native I_SA_ in CG cells.

### Reconstitution of native I_SA_ from CG cells requires not only the appropriate DPLP variant but also the appropriate KChIP variant

To the best of our knowledge, this study represents the first time a reconstituted I_SA_ channel has been assembled using a combination of native subunits for the heterotrimeric complex found in a specific neuron type and compared side-by-side with its native counterpart. Previous reconstitution experiments had made comparisons to published results without focusing on a particular neuron [16], [23] or without knowledge of the complete variant expression profile [20], [26], [29]. Care was taken in recording I_SA_ from CG cells in slice preparation in the whole-cell patch configuration, and the results obtained were minimally affected by series resistance and space clamp issues due to the compact electrical properties of these neurons. Finally, measurements were also made on dissociated neurons and the results are essentially identical to what we have seen in slices (data not shown).

The values we obtained for various I_SA_ functional properties are comparable to those reported in the literature. For activation, the midpoint and slope factor values are similar to the previously reported values, where V_0.5a_ ranged from −15 to −27 mV and S_a_ ranged from 19 to 22 mV/e-fold, depending on the location of the cell [29], [43]. For inactivation, the published inactivation midpoint and slope factor values for steady-state inactivation were also similar to our values, with V_0.5i_ ranging from −74 to −82 mV and S_i_ ranging from 5.9 to 8.4 mV/e-fold [29], [44]. The kinetics of inactivation has been described previously by exponential fitting (τ-1 = 7–20 ms, τ-2 = 60–250 ms, at ≥−10 mV) [38], [39] as well as half-inactivation times (t_0.5_ = ∼25 ms at +40 mV), and the measurements by both methods are in general agreement with our results.

The functional properties of heterologous channel complexes composed of Kv4.2, KChIP3a, DPP6a, and DPP6K (DPP6a∶DPP6K at 1∶2 ratio) closely approximate those of native I_SA_ channel. The most marked differences between the reconstituted and native channels are in the midpoint and slope factor of steady-state inactivation. One possible explanation for the detected difference is that the recorded native I_SA_ was contributed mostly by channel complexes containing Kv4.3 instead of Kv4.2. Both Kv4.2 and Kv4.3 transcripts and proteins are expressed in the granule cell layer of the cerebellum but with reciprocal rostrocaudal gradients, where Kv4.2 level is highest at the anterior lobules and Kv4.3 level is highest at the posterior lobules [33], [36], [45]. Consistent with the Kv4.2 channel complex, the steady-state inactivation of reconstituted channels are indeed more similar to of I_SA_ recorded by Amarillo and colleagues from the anterior lobules than from the posterior lobe (anterior: V_0.5i_ = −74 mV, S_i_ = 6.9 mV; posterior: V_0.5i_ = −82 mV, S_i_ = 5.9) [29]. We have tested the effect of replacing Kv4.2 with Kv4.3 and mixing Kv4.2 and Kv4.3 at a 1∶1 ratio in the ternary complex with DPP6a and DPP6K at 1∶2 ratio, and the results show that, compared to Kv4.2, Kv4.3 mediates a more hyperpolarized steady-state inactivation with a midpoint more similar to the native I_SA_ value from posterior lobes (data not shown). However, the slope factor becomes even more reduced and more different from the native I_SA_ value, and the more hyperpolarized inactivation midpoint appears to be part of a general alteration in voltage dependence that dramatically decreases the match between native and reconstituted currents in other functional properties, as exemplified by the midpoint of activation that is ∼10 mV more hyperpolarized compared to Kv4.2-based complex. Thus, substitution of Kv4.3 for Kv4.2 alone apparently cannot explain the differences between the steady-state inactivation of native and reconstituted channels.

Alternatively, the differences in steady-state inactivation may be due to the influence of additional modulatory or associated proteins. A potential candidate is the T-type calcium channel, which according to published reports can interact with Kv4.2 ternary channel complexes and specifically modulate steady-state inactivation by rightward shifting the inactivation midpoint [46], [47]. CG cells reportedly do not have T-type calcium currents [48], and the cerebellum as a whole has heterogeneous distribution of the transcripts for T-type calcium channel α-subunits (Cav3.1, Cav3.2, Cav3.3), with undetectable to moderate levels throughout the cerebellum except the caudal lobules [49]. On the other hand, *Xenopus* oocyte reportedly expresses T-type calcium current that is enhanced by the introduction of accessory beta-subunit [50]. More detailed experiments will be necessary to determine whether the endogenous T-type calcium channels underlie the difference between native and reconstituted I_SA_ channels.

Our results also show that the precise variant or type of KChIP present also has influence over the I_SA_ channel properties. When KChIP3a is substituted by KChIP4bL in the complex, the reconstituted channel no longer resembles the native channel in all parameters examined, notably with the kinetics of recovery becoming significantly slower and the G_p_-V curve shifting 10 mV in the hyperpolarizing direction. The reason behind the effects of different KChIP N-terminal variants in the ternary complex is unclear; however, it is likely to be complex and may derive from different N-terminal structures such as the presence of transmembrane segments and fatty acid modification (palmitoylation, myristylation) [12], [51]. Nevertheless, our results suggest that neurons may precisely control not only the expression of DPLP variants but also those of KChIP variants to generate distinct I_SA_ properties.

### Limitations to the reconstitution of I_SA_ channels

In this paper, we have simplified the composition of expressed subunits down to Kv4.2, DPP6a, and DPP6K (at their experimentally determined ratio) with either KChIP3a or KChIP4bL, even though a large number of I_SA_ subunits are reportedly expressed in CG cells, including Kv4.2, Kv4.3, KChIP3a, KChIP1, KChIP3a, KChIP3b (KChIP3x), KChIP4a, KChIP4b, KChIP4bL,KChIP4d, KChIP4e, DPP6a, DPP6S, and DPP6K [19], [24], [26], [28], [33], [34], [35], [36], [45]. This was done because our studies on DPP6S and DPP6L have not noted unique functional effects of these variants, even in the ternary complex [23]. In addition, other than our measurements on the expression of DPP6 variants in this paper, the relative ratios for expression of different subunits in CG cells are unknown. Also, our simplification was conducted with the following considerations. First, the selection of Kv4.2 rather than Kv4.3 likely only moderately alter the final outcome, as (1) Kv4.2 and Kv4.3 properties are quite similar when expressed in oocytes (data not shown) and (2) when bound by KChIPs, Kv4.2 and Kv4.3 properties normalize [9]. Second, analysis was conducted using KChIP3a and KChIP4bL because (1) KChIP1 expression is significantly lower than those of KChIP3 and KChIP4 based on immunohistochemistry [36], and (2) RT-PCR results have shown that KChIP3a expression is significantly greater than that of KChIP3b/3x [35]. Third, DPP6S and DPP6L are functionally similar [12] and only comprise 25% of total DPP6 transcript in cerebellar granule neurons. In the end, our finding that, with the exception of steady-state inactivation, all major biophysical parameters between the reconstituted channels and the native channels provide good match is a good indicator of the appropriateness of our reconstitution.

### Physiological relevance of our studies

The kinetic and voltage-dependent properties of I_SA_ are important determinants for the excitability and firing properties of neurons, and our study suggests that these I_SA_ properties are dependent on the precise KChIP and DPLP variants that are expressed in specific neurons. In CG cells, multi-exponential inactivation and recovery from inactivation reflect the presence of DPP6a and DPP6K variants in the ternary channel complex. However, in other cell types where DPP6K is expressed without DPP6a, DPP6K may have even greater influence over I_SA_ properties. For example, Nadal et al. (2006) reported that most of the DPP6 transcripts in the globus pallidus are contributed by DPP6K. The I_SA_ from globus pallidus shows recovery from inactivation that is bi-exponential at −95 to −100 mV, with a fast time constant of ∼60 ms and a slow time constant of greater than 360 ms [52], [53]. Our results of recovery from inactivation for Kv4.2+KChIP3a+DPP6K are similar to those of globus pallidus I_SA_, with a double exponential time course with 57±10 ms and 294±26 ms time constant for fast and slow components.

Variants of auxiliary subunits are regulated by post-translational modifications such as phosphorylation and oxidation/reduction reactions, and the presence of specific variant may confer such regulatory effect. For example, Kvbeta1 confers rapid N-type inactivation to non-inactivating Kv1 channels, and oxidation and reduction of a cysteine at the Kvbeta1 N-terminus (C7) regulates this N-type inactivation [54]. Our results show that the DPP6a variant is a major contributor to DPP6 in CG cells, and preliminary results from our lab also show that N-type inactivation conferred by DPP6a is also subject to regulation by oxidation/reduction of an N-terminal cysteine residue (unpublished data). Interestingly, oxidative stress is thought to be involved in apoptosis, and modulation of I_SA_ gating properties has been shown to confer neuroprotective effects against apoptosis in CG cells [55], [56]. An acceleration of apoptosis is a proposed factor in the causes of amyotrophic lateral sclerosis (ALS), a neurodegenerative disease associated with DPP6 and caused by degeneration of motor neurons [57].

In conclusion, our results identify both DPP6a and DPP6K as important functional modulators of the native I_SA_ channel in CG cells. For the initial phase of inactivation and recovery from short depolarizations, the rapid N-type inactivation domain of DPP6a is expected to dominate. However, for setting of the inactivation midpoint and inactivation responses to fluctuations in resting membrane potential, DPP6K is expected to play a critical role because the N-terminus of this variant contains a potent modulator of channel recovery from slower conformationally driven inactivated states. Finally, analysis of the specific effects of different KChIP proteins in the ternary channel complex suggests that KChIP3a is likely a key contributor in shaping the properties of the CG cell native I_SA_ channel.

## Supporting Information

Figure S1 **Expression pattern of DPP6a mRNA in p12 rat brain.** Autoradiography of sagittal section and coronal sections of a p12 rat brain hybridized with ^35^S-labeled DPP6a antisense and sense probes. Ctx, cortex; Hip, hippocampus; Crb, cerebellum; SC, superior colliculus; IC, inferior colliculus; RN, red nucleus; PG, pontine grey; PIR, piriform cortex, Th, thalamus; MA, magnocellular preoptic nucleus; CM, central medial nucleus; PCN, paracentral nucleus thalamus; MDm, central medial nucleus; bic, brachium of the inferior colliculus.
(TIF)
