# Supplementary figures and images for: Incorporation of DPP6a and DPP6K Variants in Ternary Kv4 Channel Complex Reconstitutes Properties of A-type K Current in Rat Cerebellar Granule Cells
Source: PLoS One. 2012 Jun 4;7(6):e38205. doi: 10.1371/journal.pone.0038205 (PMC3366920; doi:10.1371/journal.pone.0038205)

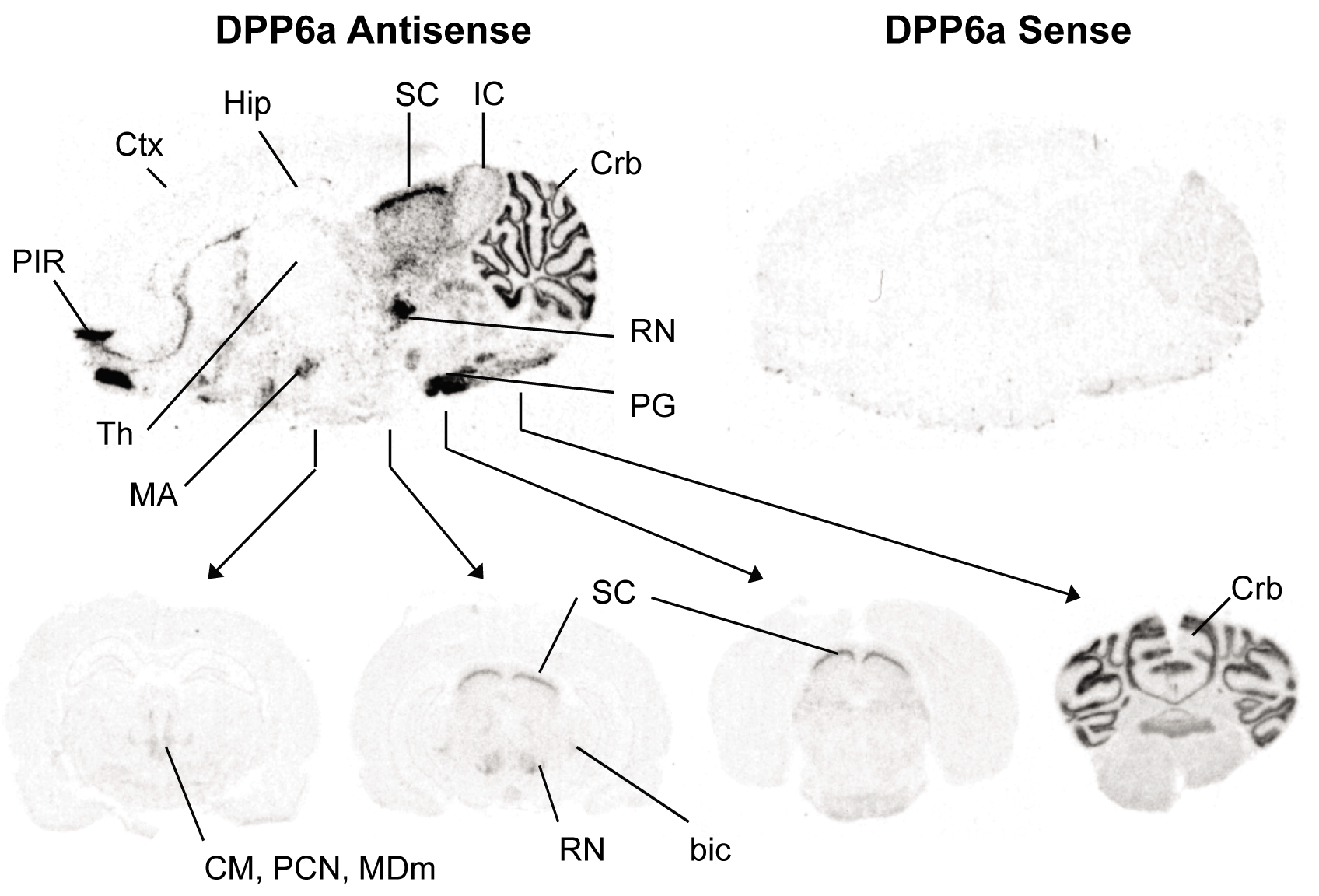

Supplement: Figure S1 — Expression pattern of DPP6a mRNA in p12 rat brain. Autoradiography of sagittal section and coronal sections of a p12 rat brain hybridized with 35S-labeled DPP6a antisense and sense probes. Ctx, cortex; Hip, hippocampus; Crb, cerebellum; SC, superior colliculus; IC, inferior colliculus; RN, red nucleus; PG, pontine grey; PIR, piriform cortex, Th, thalamus; MA, magnocellular preoptic nucleus; CM, central medial nucleus; PCN, paracentral nucleus thalamus; MDm, central medial nucleus; bic, brachium of the inferior colliculus. (TIF) [file pone.0038205.s001.tif]
